# *Ocular Albinism Type 1* Regulates Melanogenesis in Mouse Melanocytes

**DOI:** 10.3390/ijms17101596

**Published:** 2016-09-27

**Authors:** Tianzhi Chen, Haidong Wang, Yu Liu, Bingling Zhao, Yuanyuan Zhao, Ruiwen Fan, Pengchao Wang, Changsheng Dong

**Affiliations:** 1 College of Animal Science and Veterinary Medicine, Shanxi Agricultural University, Jinzhong 030600, China; chentianzhi15@163.com (T.C.); whd1232123@163.com (H.W.); 18404981230@163.com (B.Z.); ruiwenfan@163.com (R.F.); wangsh0402@163.com (P.W.); 2 College of Animal Science and Technology, Northeast Agricultural University, Harbin 150000, China; m15046093176@163.com; 3 Wujiang River Institute of Agricultural & Forestry Economic, Tongren University, Tongren 554300, China; 84840293@163.com

**Keywords:** *ocular albinism type 1* (*OA1*), *microphthalmia-associated transcription factor* (*MITF*), melanosome, coat color

## Abstract

To investigate whether *ocular albinism type 1* (*OA1*) is differentially expressed in the skin of mice with different coat colors and to determine its correlation with coat color establishment in mouse, the expression patterns and tissue distribution characterization of *OA1* in the skin of mice with different coat colors were qualitatively and quantitatively analyzed by real-time quantitative PCR (qRT-PCR), immunofluorescence staining and Western blot. The qRT-PCR analysis revealed that *OA1* mRNA was expressed in all mice skin samples tested, with the highest expression level in brown skin, a moderate expression level in black skin and the lowest expression level in gray skin. Positive OA1 protein bands were also detected in all skin samples by Western blot analysis. The relative expression levels of OA1 protein in both black and brown skin were significantly higher than that in gray skin, but there was no significant difference between black and brown mice. Immunofluorescence assays revealed that OA1 was mainly expressed in the hair follicle matrix, the inner and outer root sheath in the skin tissues with different coat colors. To get further insight into the important role of *OA1* in the melanocytes’ pigmentation, we transfected the *OA1* into mouse melanocytes and then detected the relative expression levels of pigmentation-related gene. Simultaneously, we tested the melanin content of melanocytes. As a result, the overexpression of *OA1* significantly increased the expression levels of *microphthalmia-associated transcription factor* (*MITF*), *tyrosinase* (*TYR*), *tyrosinase-related protein 1 *(*TRP1*) and *premelanosome protein *(*PMEL*). However, the *tyrosinase-related protein 2* (*TRP2*) level was attenuated. By contrast, the level of *glycoprotein non-metastatic melanoma protein b* (*GPNMB*) was unaffected by *OA1* overexpression. Furthermore, we observed a significant increase in melanin content in mouse melanocyte transfected *OA1*. Therefore, we propose that *OA1* may participate in the formation of coat color by regulating the level of *MITF* and the number, size, motility and maturation of melanosome.

## 1. Introduction

Melanosomes are the melanocyte-specific organelles within which melanin pigments are synthesized, deposited and transported. Melanosomes derive from endosomal precursors and subsequently go through a series of morphologically- and functionally-defined stages [1]. The first two stages lack pigment, but are characterized by intraluminal matrix fibrils that start to form in the stage I melanosome and are completed in the ellipsoidal stage II melanosome. Deposition of melanin along the matrix fibrils is initiated in the stage III melanosome until all internal structures are obscured in the stage IV melanosome [2]. To date, proteomic analysis of melanosomes has been found about 1500 proteins appearing in all stages of melanosomes [3]. Twelve of these proteins are specifically localized in melanosomes, including tyrosinase (TYR), tyrosinase-related protein 1 (Tyrp1/TRP1), tyrosinase-related protein 2 (Tyrp2/TRP2), ocular albinism type 1 (OA1), melanoma antigen recognized by T-cells (MART-1/MLANA), premelanosome protein (PMEL), vesicle amine transport 1 (VAT-1), oculospanin, syntenin, glycoprotein non-metastatic melanoma protein b (GPNMB), coiled-coil-helix-coiled-coil-helix domain containing 3 (CHCHD3) and flotillin [4]. The function of three melanogenesis-controlling enzymes are well known to us [5], but the roles of other melanosomal proteins are partially understood, such as OA1 [6,7].

*Ocular albinism type 1* was initially positionally cloned from the distal short arm of the X chromosome by Bassi [8]. This gene encodes a 404-amino acid internal membrane glycoprotein, named OA1, also identified as G protein-coupled receptor 143 (GPR143) [9]. Previous work displayed that OA1 exhibits structural properties of G protein-coupled receptors (GPCRs) [10,11,12]. Additionally, OA1 shared two fundamental homologies with GPCRs, activating heterotrimeric G proteins and interacting with arrestins [13]. However, differently from a canonical GPCR, OA1 is predominantly localized to the melanosomal membrane rather than the plasma membrane, with its C terminus toward the cytoplasm and with the N terminus toward the lumen of organelle [12]. Based on the localization, a putative ligand should bind OA1 on the luminal side of melanosome compared with receptors at the cell surface. Lopez et al. propose that L-DOPA is an endogenous ligand for OA1 by using a heterologous expression system based on OA1 overexpression in Chinese hamster ovary (CHO) cells [14,15,16,17]. Proteomic analysis of early melanosomes obtained from human MNT1 cells identifies OA1 as a component of these organelles [4]. All of these results suggest that OA1 may play an important role in regulating the signal process related to melanosome function. 

Networking analysis of genes by the GeneMANIA algorithm revealed that the *TYR*, *oculocutaneous albinism II* (*OCA2*), *OA1*, *microphthalmia-associated transcription factor* (*MITF*) and cbp/p300 interacting transactivator with glu/asp rich carboxy-terminal domain 1 (*CITED1*) genes were involved in the biological process of developmental pigmentation, whereas the *TYR*, *OCA2*, *OA1*, *dopachrome tautomerase* (*DCT*) and *CITED1* genes were implicated in the major biological process of biosynthetic pigment and the pigment metabolic process. The pigmentation process was involved in the interaction of the *TYR*, *OCA2* and *OA1* along with the *MITF*, *PMEL* and *CITED1* genes [18]. The transcriptional regulation of *OA1* was controlled by MITF, which belongs to the basic helix-loop-helix–leucine zipper (bHLH-LZ) factor family [19]. To date, MITF has been reported to activate more than 25 pigmentation-related genes, including *TYR*, *tyrosinase-related protein 1 *(*TRP1*), *tyrosinase-related protein 2* (*TRP2*), *MART-1*, *melanocortin 1 receptor* (*MC1R*), *endothelin receptor type b* (*EDNRB*), *RAB27A*, *PMEL*, *GPNMB*, *solute carrier family 45 member 2* (*SLC45A2/MATP*), *melastatin*, and so forth. Therefore, MITF has been regarded as an essential regulator for melanocyte survival, proliferation, development and the expression of melanogenic proteins [20]. In addition, investigations showed that the loss of OA1 function would dramatically reduce the expression of *MITF*, although the levels are still sufficient to sustain the survival and differentiated status of the pigmented cell [21]. Together, we ask whether the OA1 will participate in the establishment of coat color by regulating the level of MITF.

## 2. Results

### 2.1. Expression Profile of Ocular Albinism Type 1 (OA1) mRNA in Mice Skin Samples

**Figure 1:**
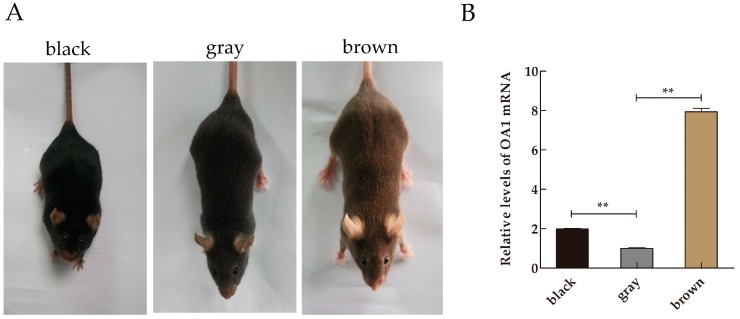
The *ocular albinism type 1* (*OA1*) mRNA analysis. (**A**) Photographs of C57BL/6J mice with a variety of coat colors; (**B**) Relative expression levels of *OA1* mRNA in the mice skin samples of different coat colors. The abundance of *OA1* was normalized relative to the abundance of *β-actin*. Data are shown as the mean ± standard errors (*n* = 3 each), ** *p *< 0.01.

*OA1* mRNA expression levels in the skin of mice with different coat colors are shown in Figure 1B. The qRT-PCR results showed that the relative expression levels of *OA1* mRNA were 1.9844 ± 0.0554** (*p *< 0.01) in black skin, 7.9394 ± 0.2998** (*p *< 0.01) in brown skin and 1.002 ± 0.0742 in the gray skin. These differences correspond to 1.98- and 7.94-fold increases in *OA1* mRNA in black skin and in brown skin relative to gray skin, respectively. The increases in *OA1* expression in black and brown mice skin compared with gray mice skin were significantly different.

### 2.2. Protein Expression of OA1 in Mice Skin Samples

**Figure 2:**
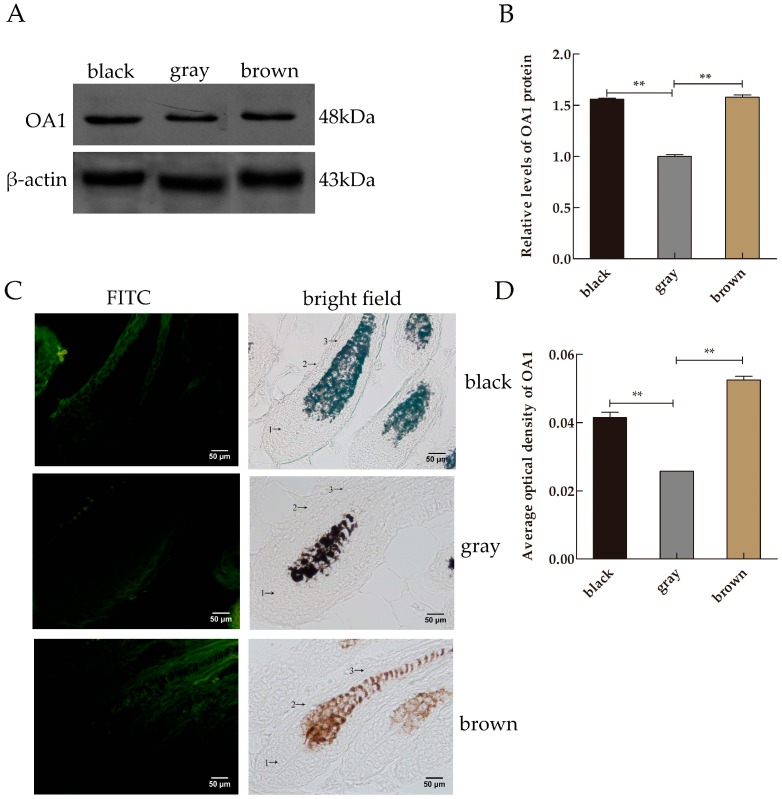
The OA1 protein analysis. (**A**) Western blot of lysates collected from the skin samples of mice with different coat colors were conducted using OA1 as the primary antibody. β-Actin was used as the loading control; (**B**) Relative expression levels of OA1 protein in the skin of mice with different coat colors. Data are shown as the mean ± standard errors (*n* = 3 each), ** *p *< 0.01; (**C**) Localization of OA1 protein in hair follicle. The immunofluorescence assay was used to determine the location of OA1 among different coat colors of mice skin. **1**: hair follicle matrix; **2**: outer root sheath; **3**: inner root sheath; (**D**) Average optical density analysis of OA1 in mice skin with different hair color. Data are shown as the mean ± standard errors (*n* = 3 each), ** *p *< 0.01.

Western blot was performed to further analyze the expression level of OA1 protein in the mice skin tissues with different coat colors. Western blot analysis showed that the total proteins from skin samples of mice with different coat colors were positively immunoreactive to the OA1 anti-rabbit polyclonal antibody, with the target band located at the position of 48 kDa (Figure 2A). Protein band analysis showed that the relative expression levels of OA1 protein were 0.5143 ± 0.0074** (*p *< 0.01) in black skin, 0.5212 ± 0.0124** (*p *< 0.01) in brown skin and 0.3300 ± 0.0100 in gray skin. These differences correspond to 1.56- and 1.58-fold higher expression in black and brown skin compared with gray skin, respectively. The protein expression levels of OA1 were significantly affected in different colors (Figure 2B).

### 2.3. Distribution and Expression of OA1 in Mice Skin Samples

The immunofluorescence assay was performed to determine the location of OA1 in mice of various coat colors. OA1 was found in the inner and outer root sheath, hair follicle matrix and dermal papilla in skin. Different levels and distributions of OA1 were found in various coat colors. In black mice, OA1 staining was strongly positive, especially in the dermal papilla, follicle matrix and outer root sheath melanocytes. However, in brown and gray mice, OA1 was mainly located in the outer root sheath of hair follicle, and it was weakly positive in gray mice (Figure 2C). No positive expression was observed in the negative control (no primary or secondary antibody added). The average optical density (OD) values for OA1 were 0.0418 ± 0.0037** (*p *< 0.01) in black skin, 0.0525 ± 0.0019* (*p *< 0.05) in brown skin, respectively, and 0.0257 ± 0.0010 in gray skin. These values indicate that OA1 is expressed at 1.62- and 2.0-fold higher levels in black and brown skin compared with gray skin, respectively (Figure 2D).

### 2.4. OA1 Controls Not Only Melanosome Structural Protein but Also the Melanin Synthesis in an MITF-Dependent Fashion

**Figure 3:**
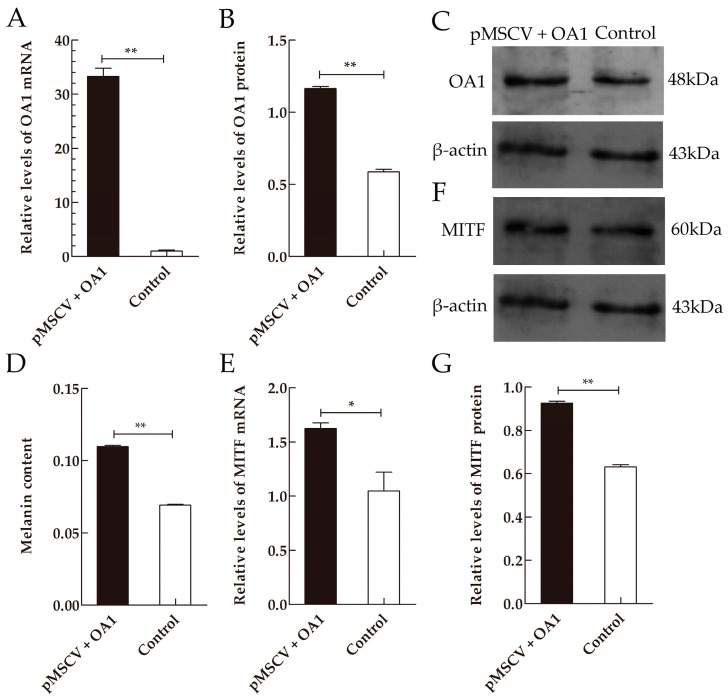
The overexpression of OA1 affects the melanin content in an MITF-dependent fashion. Mice *OA1* was PCR amplified and then subcloned into the pMSCV PIG vector with Xho I and EcoR I restriction sites. The pMSCV-OA1-GFP plasmid or empty vector was transfected into the mouse melanocytes. The cells were harvested to extract total RNA and total protein. The expression levels of *OA1* were qualitatively and quantitatively analyzed by qRT-PCR and Western blot. (**A**) Melanin contents in melanocytes transfected with *OA1*, as well as the control; (**B**–**D**) transfection efficiency of *OA1* in mouse melanocytes; (**E**–**G**) OA1 overexpression influences *microphthalmia-associated transcription factor* (*MITF*) mRNA and protein levels in mouse melanocytes. Data are shown as the mean ± standard errors (*n* = 3 each), * *p* < 0.05, ** *p* < 0.01.

To determine the effect of OA1 on the melanogenesis, we transfected mice melanocytes with the plasmid encoding OA1. There are two groups in this experiment, including the vector-GFP-OA1 group and the mock-vector-GFP group. The qRT-PCR and Western blot analysis showed that the relative expression levels of *OA1* mRNA and protein in the OA1-transfected group were 31.90-fold and 1.98-fold higher than that in the control group, respectively (Figure 3A–C). All of these results suggest that *OA1* has been transfected into melanocytes efficiently.

To confirm the effect of OA1 on the melanin biosynthesis in melanocytes, the melanin content in cells transfected with pMSCV-GFP-OA1 or pMSCV-GFP was measured. The results demonstrated that the melanin content in the OA1 overexpression melanocytes was 1.59-fold higher than that in the control group (Figure 3D).

Our data indicate that *OA1* presents a different expression in mice skin of various coat colors. Therefore, we suspect that OA1 might participate in the formation of coat color, possibly by regulating the expression of some melanosomal protein in an MITF-dependent fashion [22]. To verify this hypothesis, we measured the levels of *MITF*, *TYR*, *TRP1*, *TRP2*, *PMEL* and *GPNMB*. *MITF*, encoding an integral transcriptional regulator in melanocytes, has been mapped to chromosome 6 in mice. It controls the transcription levels of pigmentation-associated gene and the differentiation, proliferation and survival of melanocytes. TYR is a type I membrane glycoprotein with seven potential *N*-glycosylation sites [23]. TYR, the first rate-limiting enzyme in melanin synthesis, catalyzes the conversion of l-tyrosine to dopaquinone and the oxidation of 3,4-dihydroxyphenylalanine (DOPA) back to dopaquinone [24]. TRP1 is a 5,6-dihydroxyindole-2-carboxylic acid (DHICA) oxidase, which promotes the oxidation and polymerization of DHICA monomers into melanin [25]. TRP2 possesses dopachrome tautomerase (DCT) activity, which enables a rapid conversion of dopachrome (DC) to DHICA [26,27]. PMEL, also known as PMEL17, is an internal membrane glycoprotein that constitutes the fibrillar matrix of melanosome [28,29,30]. GPNMB is a highly glycosylated type I transmembrane protein with a high level of structural homology to PMEL [31,32].

**Figure 4:**
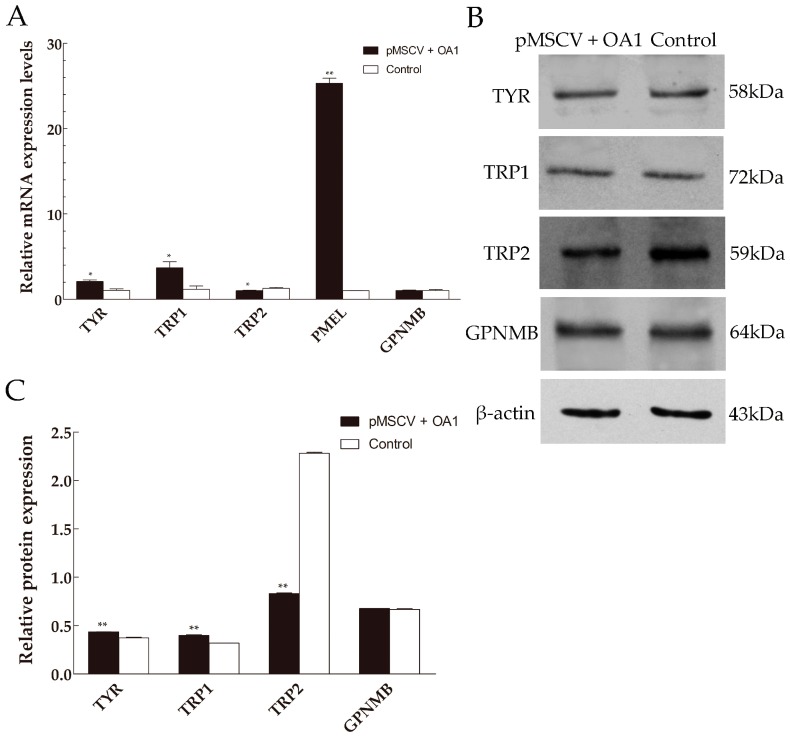
The overexpression of OA1 affects melanosomal proteins in mouse melanocytes. (**A**) The pigmentation-related gene mRNA levels were measured by qRT-PCR; (**B**,**C**) Western blot of cell lysates probed with primary antibody against the TYR, TRP1, TRP2 and GPNMB. β-actin was used as a protein loading control. Data are shown as the mean ± standard errors (*n* = 3 each), * *p* < 0.05, ** *p* < 0.01.

A previous study found that the expression level of *MITF* reduced correlating with the reduced *OA1* expression in *OA1*^−/−^ or shRNA-depleted melanocytes [22]. Consistently, we observed an increase in *MITF* mRNA and protein levels when *OA1* was overexpressed in the mouse melanocytes (Figure 3E–G). These results revealed that OA1 was a signaling protein controlling the *MITF* expression levels. To get further insights into the function of OA1 in melanocytes, we measured the expression levels of some pigmentation-related genes. Real-time quantitative PCR analysis demonstrated that the expression levels of *TYR*, *TRP1* and *PMEL* mRNA were upregulated in the OA1 overexpression melanocytes compared with the control (Figure 4A). Consistent with this, when we analyzed the levels of TYR and TRP1 protein by Western blot, we found that both were modestly increased (Figure 4B,C). However, compared with the control, the expression levels of *TRP2* mRNA and protein were attenuated in the OA1 overexpression melanocytes (Figure 4). By contrast, the expression levels of *GPNMB* mRNA and protein were not affected after *OA1* was transfected into the mouse melanocytes. The housekeeping protein β-actin was used as a loading control.

## 3. Discussion

The color we see in the skin, hair and eyes of mammals is largely determined by the presence and distribution of melanin. There are two main types of melanin in mammal melanocytes, including brown-black eumelanin and red-yellow pheomelanin [33]. Melanin synthesis exclusively occurs in subcellular organelles termed melanosomes within melanocytes and retinal pigment epithelium (RPE), which is mainly controlled by TYR, TRP1 and TRP2 [34,35,36,37,38]. There are four stages in the development of melanosome: unmelanized immature premelanosome in stage I and II melanosome and melanized stage III and IV melanosome [39].

Melanocytes, which are originally derived from neural crest cells in embryonic skin, are localized in epidermis, as well as in hair follicles to pigment the skin and hair, respectively [40,41,42]. In the hairy skin of mice, melanocytes in the epidermis are found only during the early weeks after birth [43]. Different from the epidermal melanocytes, melanocytes in hair follicles repeatedly proliferate and differentiate for hair pigmentation in every hair cycle. Previous work has shown that there are three morphologically- and functionally-discrete melanocytic populations in hair follicles: melanocyte stem cells, melanocyte progenitor cells and terminally-differentiated melanocytes [44]. The first two localized in the outer root sheath (ORS) of the middle and lower hair follicles express DCT and TRP1, but lack TYR, so they do not produce melanin and belong to the amelanotic melanocytes. The latter residing in the infundibulum and bulb express all enzymes necessary for the melanogenesis, so they belong to the pigmented melanocytes [45,46,47,48]. These ORS melanocytes act as a melanocyte reservoir for the repopulation of epidermal melanocytes and for repigmentation of vitiligo [49,50]. Proteomic analysis of melanosomes found that OA1 was mainly enriched in compartments highly labeled by PMEL17 that correspond to stage I and stage II premelanosomes, although it appeared broadly distributed in all stages of melanosome maturation [51]. In common with this, the immunofluorescence assay revealed that OA1 was primarily expressed in the outer root sheath of hair follicle. In addition, it was also localized in the dermal papilla and hair follicle matrix. These results further support that OA1 plays an important role in the early stages of melanosome biogenesis, as predicted previously.

Here, we demonstrated that different coat colors were associated with different levels of *OA1* expression. *OA1* mRNA and protein were expressed in all mice skin samples tested, with the highest expression level in brown mice skin, a moderate expression level in black mice skin and the lowest in gray mice skin. These observations revealed that *OA1* transcription and translation levels are high in the skin of black and brown mice and low in the skin of gray mice. Therefore, we propose that OA1 may be involved in the establishment of coat color, of which the specific mechanism is as follows. The mutation of *OA1* is responsible for the most common form of ocular albinism, in which patients exhibit a reduced number of enlarged melanosomes and visual defects [8,11,52]. OA1 performs two functions in melanogenesis, controlling the rate of melanosome biogenesis at early maturation stages and maintaining the correct melanosomal size at the final stage of the organellogenesis [53]. OA1 regulates not only melanosome biogenesis, but also the motility of melanosomes. Time-lapse video-microscopy and melanosome tracking analyses reveal that the absence of OA1 results in a significant reduction in frequent melanosomal movement along microtubules. However, this observation exclusively occurs in the presence of an intact actin cytoskeleton. Thus, the cytoskeleton represents a downstream effector of OA1 for which the receptor plays a modulatory role in melanosome motility, by switching between MT-based and AF-based systems [54,55]. Burgoyne et al. demonstrated that OA1 expression increased multivesicular endosomes’/bodies’ (MVBs) number. OA1 could inhibit MVB-lysosome fusion through affecting the motility of MVB or lysosome and the luminal pH of MVBs. A delay in lysosomal fusion might allow time for melanin deposition [56]. Taken together, we propose that OA1 may participate in the formation of coat color differences through regulating the number, size, motility and maturation of melanosomes.

OA1 controls the melanosome composition and density at early stages of melanogenesis [51]. Therefore, we thought there was a possible connection between the expression of *OA1* and *MITF*. Our data showed that the overexpression of OA1 in mouse melanocytes caused an increase in the *MITF* levels. Consistently, the expression level of *MITF* was attenuated when OA1 expression was absent [22]. These results demonstrate that OA1 could positively control MITF. The role of OA1 in regulating MITF expression participates in the α-MSH-MITF-signaling pathway [22]. The α-melanocyte-stimulating hormone (α-MSH) binding to MC1R activates adenylate cyclase, which results in the activation of cyclic adenosine monophosphate (cAMP) signaling. Additionally, then, cAMP leads to the phosphorylation of cAMP response element binding protein (CREB) that recognizes the *MITF* promoter [57].

Because OA1 activity positively leads to the regulation of MITF, we asked whether MITF-controlled genes will be affected by its modulation via OA1 [22]. A previous study found that *PMEL* was an MITF target gene in melanocytes and melanoma, with its mRNA levels being enhanced upon transfer of wt *MITF* and blocked by a dominant negative form of *MITF*, which indicated that the expression level of MITF was necessary for the *PMEL* gene transcription [58]. In addition, the expression level of *PMEL* showed an increase when MITF was re-expressed in *OA1*^−/−^ LOa1SN melanocytes [15]. We observed that the relative expression level of the structural protein *PMEL* mRNA in the OA1 overexpression melanocytes was significantly higher compared with the control. Collectively, these results suggest that *OA1* regulates *PMEL* expression via MITF. By contrast, although GPNMB, encoding a type I transmembrane protein whose transcription is dependent on MITF [59], has been identified as having a high level of structural homology with PMEL, its mRNA and protein expression levels are not changed by *OA1* expression. As we all known, loss of function of OA1 causes a decrease in pigmentation and induces the formation of enlarged aberrant melanosome harboring disorganized fibrillar structures. Ultrastructural analysis of the RPE cells reveals that the formation of macromelanosomes may be caused by abnormal growth of single melanosomes rather than the fusion of several [60]. In other words, the decrease of PMEL expression by *OA1* absence leads to the slowing down of the rate of new stage II melanosome formation [61]. Collectively, the variation of *PMEL* expression resulting from OA1 overexpression is likely mediated by MITF. In addition to the structural protein, the melanosomal enzymes are also affected by the MITF level. Our observations showed that the *TYR* and *TRP1* expression was modestly increased in the OA1 overexpression melanocytes compared with the control cells. However, the overexpression of OA1 reduced the *TRP2* expression. The transient cotransfection assay displayed that MITF overexpression transactivated the human tyrosinase promoter, as well as the *TRP-1* gene promoter, rather than the *TRP-2* promoter [62]. Taken together, OA1 not only regulates melanosomal structural protein expression, but also melanin biogenesis in melanocytes in an MITF-independent fashion.

## 4. Materials and Methods

### 4.1. Antibody

Rabbit anti-OA1 polyclonal antibody and goat anti-rabbit IgG-FITC were purchased from Santa Cruz Biotechnology, Inc. (Santa Cruz, CA, USA). Rabbit anti-TYR polyclonal antibody, rabbit anti-TRP1 polyclonal antibody, rabbit anti-TRP2 polyclonal antibody and mouse anti-MITF polyclonal antibody were purchased from Abcam (Cambridge, MA, USA). Horseradish peroxidase (HRP)-conjugated goat anti-rabbit immunoglobulin G (IgG) and HRP-conjugated goat anti-mouse IgG were purchased from CWBIO (Beijing, China). Rabbit anti-GPNMB polyclonal antibody were purchased from Proteintech group (Wuhan, China).

### 4.2. Experimental Animals and Sample Collection

This study was conducted in strict accordance with the recommendations in the Guide for the Care and Use of Laboratory Animals of the National Institutes of Health (National Research Council (US) Committee for the Update of the Guide for the Care and Use of Laboratory Animals, 27 December 2010, ISBN: 9780309154000).

Three approximately 14-day-old healthy C57BL/6 mice were randomly selected from breeds with black, gray and brown coat colors (Figure 1A). After shaving and then disinfection, three pieces of dorsal skin tissue were collected from each mouse using a skin collector, of which two samples were frozen and stored in liquid nitrogen for total RNA and total protein extraction. The remaining piece of skin was fixed in Bouin’s solution to prepare paraffin sections for immunofluorescence staining.

### 4.3. Cell Culture and Transfection

Mouse melanocytes (derived from C57BL/6J black mice) were established in the laboratory of alpaca biology, College of Animal Science and Technology, Shanxi Agricultural University (Jinzhong, China) [63]. Mouse melanocytes were cultured in melanocyte medium (MelM) (ScienCell Research Laboratories, Carlsbad, CA, USA) supplemented with 1% melanocyte growth supplement (MelGS) and 1% penicillin-streptomycin and 0.5% fetal bovine serum at 37 °C in a humidified 5% CO_2_ atmosphere.

The mice *OA1* gene was PCR amplified and then was cloned into the pMSCV PIG vector (Addgene, Cambridge, MA, USA) with Xho I and EcoR I restriction sites. Mouse melanocytes were transfected using Lipofectamine 2000 reagent (Invitrogen, Carlsbad, CA, USA) according to the manufacturer’s guidelines. Then, we harvested the cells and extracted the total RNA and total protein.

### 4.4. Melanin Content Measurement

Seventy-two hours after transfection, mouse melanocytes were collected and washed in phosphate buffer saline (PBS) (Solarbio, Beijing, China) 3 times. The cells were resuspended in PBS and counted using an automated cell counter (Bio-Rad, Hercules, CA, USA). The cells were centrifuged at 1000 rpm for 10 min at 4 °C, and 1 mL of 1 mol/L NaOH was added followed by mixing and incubation at 80 °C for 30 min. Then, the melanin content was measured at 475 nm. The melanin content was homogenized to the empty vector group.

### 4.5. RNA Extraction and qRT-PCR Analysis

Total RNA was extracted from mice skin tissues or melanocytes using TRIzol reagent (TAKARA, Dalian, China). The concentration and integrity of total RNA were determined using the NanoDrop 1000 spectrophotometer (NanoDrop, Wilmington, NA, USA) and identified by 1% agarose gel electrophoresis, respectively. Complementary (c) DNA synthesis was performed using the PrimeScript^TM^ RT regent Kit (TAKARA, Dalian, China) following the manufacturer’s instructions. The real-time quantitative PCR was performed using SYBR^®^Premix Ex TaqTMII (Tli RNaseH Plus) (TAKARA, Dalian, China). All qRT-PCR reactions were performed in triplicate on the StepOne PlusTM Real-Time PCR System (Applied Biosystems, Inc., Foster City, CA, USA). Quantification of *OA1* transcript abundance was performed using the comparative threshold cycle (*C*_t_) method. Transcript levels of gene were normalized relative to those of the internal control *β-actin*. The primer details are shown in Table 1.

### 4.6. Western Blotting

Total protein from mice skin tissues or melanocytes was extracted using total protein extraction reagent (Beyotime, Shanghai, China) according to the manufacturer’s instructions, and the concentration was measured using a nucleic acid/protein analyzer. Two hundred micrograms of protein lysate from each sample were size-separated by SDS-PAGE electrophoresis and transferred onto nitrocellulose filter membranes (Boster, Wuhan, China). The membranes were blocked in 5% skimmed milk powder (Boster, Wuhan, China) at room temperature for 1 h and then were probed with the primary antibody diluted in Tris-buffered saline-Tween (TBST) overnight at 4 °C. The next day, the membranes were washed 3 times in TBST for 10 min each and incubated with HRP-conjugated second antibody (1:10,000 (*v*/*v*), Boster, Wuhan, China) at 37 °C with horizontal shaking for 1 h. Subsequently, the membranes were washed 6 times in TBST for 5 min each, and the proteins were visualized via a super ECL chemiluminescence solution (Boster, Wuhan, China). The Western blot results were analyzed using Image-ProPlus 6.0 software (Olympus, Hatayaga, Japan) to measure the area and gray value for each target band. The target protein expression level was normalized relative to the corresponding internal reference level in each lane.

### 4.7. Localization of OA1 by Immunofluorescence Staining

Paraffin-embedded tissues were sectioned, dewaxed for hydration, incubated in 3% H_2_O_2_ (Boster, Wuhan, China) at 37 °C for 10 min and then washed 3 times in PBS with shaking for 2 min each. Next, the tissues were blocked with 10% goat serum (Boster, Wuhan, China) for 30 min at room temperate. The excess liquid was then shaken off, and the OA1 anti-rabbit polyclonal antibody (1:100 diluted in PBS) was added dropwise, with no treatment in the negative control. The samples were placed at 4 °C overnight, re-warmed at 37 °C for 30 min the next day and washed 3 times in PBS (3 min per wash). Subsequently, FITC-labeled goat anti-rabbit IgG secondary antibody (1:200 diluted in PBS) was then added dropwise, followed by incubation at 37 °C for 1 h and 6 times in PBS (5 min per wash). The slices were mounted with the anti-fluorescent mounting media and observed under a fluorescence microscope.

### 4.8. Statistical Analysis

All experiments were carried out in triplicate. Values are presented as the mean ± SD. Statistical analysis was performed by GraphPad Prism 5.0 software (GraphPad Software Inc., La Jolla, CA, USA). Univariate analysis of variance was performed using SPSS 19.0 software (IBM, Armonk, NY, USA), and *p*-values <0.05 were considered statistically significant and those <0.01 as highly significant.

## 5. Conclusions

**Figure 5:**
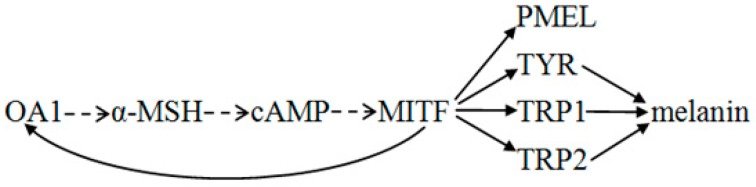
Potential pathway of OA1 in regulating melanogenesis in mouse melanocytes. Dotted arrows—to be verified; solid arrows—verified.

In this study, we found that OA1 was detectably expressed in mice skin of different coat colors, though at significantly different levels. Additionally, OA1 was mainly located in the hair follicle matrix, the inner and outer root sheath in the mice skin tissues with different coat colors. When *OA1* was overexpressed in mouse melanocytes, the expression levels of *MITF*, *TYR*, *TRP1* and *PMEL* were upregulated, and the expression level of *TRP2* was downregulated. By contrast, the levels of *GPNMB* mRNA and protein were unaffected in melanocyte transfected *OA1* compared with the control. Taken together, we propose that OA1 may participate in the formation of coat color by regulating the level of MITF (Figure 5) and the number, size, motility and maturation of melanosomes.

## Figures and Tables

**Table 1:** Primers used in this study.

Primer Name	Primer Sequence 5’→3’	PCR Production (bp)
Mus-*OA1*-F-XhoI	CCGCTCGAGGCCACCATGGCCTCCCCGCGCCT	1218
Mus-*OA1*-R-EcoRI	CGGAATTCTCAGAGTTCCCCCTGGGCTTG	
Mus-*OA1*-F	ATCAGGGCGTCGATCTGTTG	193
Mus-*OA1*-R	AGCAGGCCAAATGTCTGTTG	
Mus-*MITF*-F	AGGACCTTGAAAACCGACAG	115
Mus-*MITF*-R	GTGGATGGGATAAGGGAAAG	
Mus-*TYR*-F	ACTTACTCAGCCCAGCATCC	109
Mus-*TYR*-R	AGTGGTCCCTCAGGTGTTCC	
Mus-*TRP1*-F	CTTGGAGGTCCGTGTATTTG	223
Mus-*TRP1*-R	GACCGCATCAGTGAAAGTGT	
Mus-*TRP2*-F	CCAACGCTGATTAGTCGGA	213
Mus-*TRP2*-R	GAAGAAGGGAGGGCTGTCA	
Mus-*GPNMB*-F	GGGCATACATTCCCATCTCG	215
Mus-*GPNMB*-R	AGTGTTGTCCCCAAAGTTCCA	
Mus-*PMEL*-F	AGTGTTGTCCCCAAAGTTCCA	171
Mus-*PMEL*-R	AGGAGCGGGCTGTTTTCT	
Mus-*β-actin*-F	TTGCTGACAGGATGCAGAAG	140
Mus-*β-actin*-R	TTGCTGACAGGATGCAGAAG	

## References

[1] Seiji M., Fitzpatrick T.B., Simpson R.T., Birbeck M.S. (1963). Chemical composition and terminology of specialized organelles (melanosomes and melanin granules) in mammalian melanocytes. Nature.

[2] Hearing V.J. (2005). Biogenesis of pigment granules: A sensitive way to regulate melanocyte function. J. Dermatol. Sci..

[3] Chi A., Valencia J.C., Hu Z.Z., Watabe H., Yamaguchi H., Mangini N.J., Huang H., Canfield V.A., Cheng K.C., Yang F. (2006). Proteomic and bioinformatic characterization of the biogenesis and function of melanosomes. J. Proteome Res..

[4] Basrur V., Yang F., Kushimoto T., Higashimoto Y., Yasumoto K., Valencia J., Muller J., Vieira W.D., Watabe H., Shabanowitz J. (2003). Proteomic analysis of early melanosomes: Identification of novel melanosomal proteins. J. Proteome Res..

[5] Korner A., Pawelek J. (1982). Mammalian tyrosinase catalyzes three reactions in the biosynthesis of melanin. Science.

[6] McKay B.S., Schwartz S.G. (2016). Pigmentation and macular degeneration: Is there a role for GPR143?. J. Ocul. Pharmacol. Ther..

[7] Giordano F., Simoes S., Raposo G. (2011). The ocular albinism type 1 (OA1) GPCR is ubiquitinated and its traffic requires endosomal sorting complex responsible for transport (ESCRT) function. Proc. Natl. Acad. Sci. USA.

[8] Bassi M.T., Schiaffino M.V., Renieri A., de Nigris F., Galli L., Bruttini M., Gebbia M., Bergen A.A., Lewis R.A., Ballabio A. (1995). Cloning of the gene for ocular albinism type 1 from the distal short arm of the X chromosome. Nat. Genet..

[9] Schiaffino M.V., Baschirotto C., Pellegrini G., Montalti S., Tacchetti C., De Luca M., Ballabio A. (1996). The ocular albinism type 1 gene product is a membrane glycoprotein localized to melanosomes. Proc. Natl. Acad. Sci. USA.

[10] Sone M., Orlow S.J. (2007). The ocular albinism type 1 gene product, OA1, spans intracellular membranes 7 times. Exp. Eye Res..

[11] D’Addio M., Pizzigoni A., Bassi M.T., Baschirotto C., Valetti C., Incerti B., Clementi M., de Luca M., Ballabio A., Schiaffino M.V. (2000). Defective intracellular transport and processing of OA1 is a major cause of ocular albinism type 1. Hum. Mol. Genet..

[12] Schiaffino M.V., d’Addio M., Alloni A., Baschirotto C., Valetti C., Cortese K., Puri C., Bassi M.T., Colla C., de Luca M. (1999). Ocular albinism: Evidence for a defect in an intracellular signal transduction system. Nat. Genet..

[13] Innamorati G., Piccirillo R., Bagnato P., Palmisano I., Schiaffino M.V. (2006). The melanosomal/lysosomal protein OA1 has properties of a G protein-coupled receptor. Pigment Cell Res..

[14] Lopez V.M., Decatur C.L., Stamer W.D., Lynch R.M., McKay B.S. (2008). L-DOPA is an endogenous ligand for OA1. PLoS Biol..

[15] Fukuda N., Naito S., Masukawa D., Kaneda M., Miyamoto H., Abe T., Yamashita Y., Endo I., Nakamura F., Goshima Y. (2015). Expression of ocular albinism 1 (OA1), 3, 4-dihydroxy-L-phenylalanine (DOPA) receptor, in both neuronal and non-neuronal organs. Brain Res..

[16] Masukawa D., Nakamura F., Koga M., Kamiya M., Chen S., Yamashita N., Arai N., Goshima Y. (2014). Localization of ocular albinism-1 gene product GPR143 in the rat central nervous system. Neurosci. Res..

[17] Hiroshima Y., Miyamoto H., Nakamura F., Masukawa D., Yamamoto T., Muraoka H., Kamiya M., Yamashita N., Suzuki T., Matsuzaki S. (2014). The protein Ocular Albinism 1 is the orphan GPCR GPR143 and mediates depressor and bradycardic responses to DOPA in the nucleus tractus solitarii. Br. J. Pharmacol..

[18] Kamaraj B., Gopalakrishnan C., Purohit R. (2014). In silico analysis of mirna-mediated gene regulation in OCA and OA genes. Cell Biochem. Biophys..

[19] Vetrini F., Auricchio A., Du J., Angeletti B., Fisher D.E., Ballabio A., Marigo V. (2004). The microphthalmia transcription factor (MITF) controls expression of the ocular albinism type 1 gene: Link between melanin synthesis and melanosome biogenesis. Mol. Cell. Biol..

[20] Vachtenheim J., Borovansky J. (2010). “Transcription physiology” of pigment formation in melanocytes: Central role of MITF. Exp. Dermatol..

[21] Steingrimsson E., Copeland N.G., Jenkins N.A. (2004). Melanocytes and the microphthalmia transcription factor network. Annu. Rev. Genet..

[22] Falletta P., Bagnato P., Bono M., Monticone M., Schiaffino M.V., Bennett D.C., Goding C.R., Tacchetti C., Valetti C. (2014). Melanosome-autonomous regulation of size and number: The OA1 receptor sustains PMEL expression. Pigment Cell Melanoma Res..

[23] Ujvari A., Aron R., Eisenhaure T., Cheng E., Parag H.A., Smicun Y., Halaban R., Hebert D.N. (2001). Translation rate of human tyrosinase determines its N-linked glycosylation level. J. Biol. Chem..

[24] Cooksey C.J., Garratt P.J., Land E.J., Pavel S., Ramsden C.A., Riley P.A., Smit N.P. (1997). Evidence of the indirect formation of the catecholic intermediate substrate responsible for the autoactivation kinetics of tyrosinase. J. Biol. Chem..

[25] Kobayashi T., Imokawa G., Bennett D.C., Hearing V.J. (1998). Tyrosinase stabilization by Tyrp1 (the brown locus protein). J. Biol. Chem..

[26] Jackson I.J., Chambers D.M., Tsukamoto K., Copeland N.G., Gilbert D.J., Jenkins N.A., Hearing V. (1992). A second tyrosinase-related protein, TRP-2, maps to and is mutated at the mouse slaty locus. EMBO J..

[27] Tsukamoto K., Jackson I.J., Urabe K., Montague P.M., Hearing V.J. (1992). A second tyrosinase-related protein, TRP-2, is a melanogenic enzyme termed DOPAchrome tautomerase. EMBO J..

[28] Berson J.F., Theos A.C., Harper D.C., Tenza D., Raposo G., Marks M.S. (2003). Proprotein convertase cleavage liberates a fibrillogenic fragment of a resident glycoprotein to initiate melanosome biogenesis. J. Cell Biol..

[29] Kobayashi T., Urabe K., Orlow S.J., Higashi K., Imokawa G., Kwon B.S., Potterf B., Hearing V.J. (1994). The Pmel 17/silver locus protein. Characterization and investigation of its melanogenic function. J. Biol. Chem..

[30] Theos A.C., Berson J.F., Theos S.C., Herman K.E., Harper D.C., Tenza D., Sviderskaya E.V., Lamoreux M.L., Bennett D.C., Raposo G. (2006). Dual loss of ER export and endocytic signals with altered melanosome morphology in the silver mutation of Pmel17. Mol. Biol. Cell.

[31] Hoashi T., Sato S., Yamaguchi Y., Passeron T., Tamaki K., Hearing V.J. (2010). Glycoprotein nonmetastatic melanoma protein b, a melanocytic cell marker, is a melanosome-specific and proteolytically released protein. FASEB J..

[32] Weterman M.A., Ajubi N., van Dinter I.M., Degen W.G., van Muijen G.N., Ruitter D.J., Bloemers H.P. (1995). Nmb, a novel gene, is expressed in low-metastatic human melanoma cell lines and xenografts. Int. J. Cancer.

[33] Prota G., Hu D.N., Vincensi M.R., McCormick S.A., Napolitano A. (1998). Characterization of melanins in human irides and cultured uveal melanocytes from eyes of different colors. Exp. Eye Res..

[34] Scriver C.R., Stanbury J.B., Wyngaarden J.B., Fredrickson D.S. (1997). The Metabolic and Molecular Bases of Inherited Disease.

[35] Hearing V.J. (1993). Unraveling the melanocyte. Am. J. Hum. Genet..

[36] Hearing V.J. (2000). The melanosome: The perfect model for cellular responses to the environment. Pigment Cell Res..

[37] Ito S. (2003). The IFPCS presidential lecture: A chemist’s view of melanogenesis. Pigment Cell Res..

[38] Ito S., Wakamatsu K. (2011). Human hair melanins: What we have learned and have not learned from mouse coat color pigmentation. Pigment Cell Melanoma Res..

[39] Fitzpatrick T., Hori Y., Toda K., Seiji M. (1969). Melanin 1969: Some definitions and problems. Jpn. J. Dermatol. B.

[40] Mayer T.C. (1973). The migratory pathway of neural crest cells into the skin of mouse embryos. Dev. Biol..

[41] Nishimura E.K., Yoshida H., Kunisada T., Nishikawa S.I. (1999). Regulation of E- and P-cadherin expression correlated with melanocyte migration and diversification. Dev. Biol..

[42] Rawles M.E. (1947). Origin of pigment cells from the neural crest in the mouse embryo. Physiol. Zool..

[43] Hirobe T. (1984). Histochemical survey of the distribution of the epidermal melanoblasts and melanocytes in the mouse during fetal and postnatal periods. Anat. Rec..

[44] Lin J.Y., Fisher D.E. (2007). Melanocyte biology and skin pigmentation. Nature.

[45] Horikawa T., Norris D.A., Johnson T.W., Zekman T., Dunscomb N., Bennion S.D., Jackson R.L., Morelli J.G. (1996). DOPA-negative melanocytes in the outer root sheath of human hair follicles express premelanosomal antigens but not a melanosomal antigen or the melanosome-associated glycoproteins tyrosinase, TRP-1, and TRP-2. J. Investig. Dermatol..

[46] Sarin K.Y., Artandi S.E. (2007). Aging, graying and loss of melanocyte stem cells. Stem Cell Rev..

[47] Slominski A., Wortsman J., Plonka P.M., Schallreuter K.U., Paus R., Tobin D.J. (2005). Hair follicle pigmentation. J. Investig. Dermatol..

[48] Tobin D.J., Bystryn J.C. (1996). Different populations of melanocytes are present in hair follicles and epidermis. Pigment Cell Res..

[49] Cui J., Shen L.Y., Wang G.C. (1991). Role of hair follicles in the repigmentation of vitiligo. J. Investig. Dermatol..

[50] Ortonne J.P., Pelletier N., Chabanon M., Thivolet J. (1978). Vitiligo and cutaneous epitheliomas. Ann. Dermatol. Venereol..

[51] Giordano F., Bonetti C., Surace E.M., Marigo V., Raposo G. (2009). The ocular albinism type 1 (OA1) G-protein-coupled receptor functions with MART-1 at early stages of melanogenesis to control melanosome identity and composition. Hum. Mol. Genet..

[52] O’Donnell F.E., Hambrick G.W., Green W.R., Iliff W.J., Stone D.L. (1976). X-linked ocular albinism: An oculocutaneous macromelanosomal disorder. Arch. Ophthalmol..

[53] Cortese K., Giordano F., Surace E.M., Venturi C., Ballabio A., Tacchetti C., Marigo V. (2005). The ocular albinism type 1 (OA1) gene controls melanosome maturation and size. Investig. Ophthalmol. Vis. Sci..

[54] Palmisano I., Bagnato P., Palmigiano A., Innamorati G., Rotondo G., Altimare D., Venturi C., Sviderskaya E.V., Piccirillo R., Coppola M. (2008). The ocular albinism type 1 protein, an intracellular G protein-coupled receptor, regulates melanosome transport in pigment cells. Hum. Mol. Genet..

[55] Schiaffino M.V. (2010). Signaling pathways in melanosome biogenesis and pathology. Int. J. Biochem. Cell Biol..

[56] Burgoyne T., Jolly R., Martin-Martin B., Seabra M.C., Piccirillo R., Schiaffino M.V., Futter C.E. (2013). Expression of OA1 limits the fusion of a subset of MVBs with lysosomes—A mechanism potentially involved in the initial biogenesis of melanosomes. J. Cell Sci..

[57] Busca R., Ballotti R. (2000). Cyclic AMP a key messenger in the regulation of skin pigmentation. Pigment Cell Res..

[58] Du J., Miller A.J., Widlund H.R., Horstmann M.A., Ramaswamy S., Fisher D.E. (2003). MlANA/MART1 and SILV/PMEL17/GP100 are transcriptionally regulated by MITF in melanocytes and melanoma. Am. J. Pathol..

[59] Loftus S.K., Antonellis A., Matera I., Renaud G., Baxter L.L., Reid D., Wolfsberg T.G., Chen Y., Wang C., Prasad M.K. (2009). Gpnmb is a melanoblast-expressed, MITF-dependent gene. Pigment Cell Melanoma Res..

[60] Incerti B., Cortese K., Pizzigoni A., Surace E.M., Varani S., Coppola M., Jeffery G., Seeliger M., Jaissle G., Bennett D.C. (2000). OA1 knock-out: New insights on the pathogenesis of ocular albinism type 1. Hum. Mol. Genet..

[61] Burgoyne T., O’Connor M.N., Seabra M.C., Cutler D.F., Futter C.E. (2015). Regulation of melanosome number, shape and movement in the zebrafish retinal pigment epithelium by OA1 and PMEL. J. Cell Sci..

[62] Yasumoto K., Yokoyama K., Takahashi K., Tomita Y., Shibahara S. (1997). Functional analysis of microphthalmia-associated transcription factor in pigment cell-specific transcription of the human tyrosinase family genes. J. Biol. Chem..

[63] Shi Z., Ji K., Yang S., Zhang J., Yao J., Dong C., Fan R. (2016). Biological characteristics of mouse skin melanocytes. Tissue Cell.

